# Effect of Acute and Chronic Exposure to High Altitude on the Aerobic and Anaerobic Metabolism in Rats

**DOI:** 10.1155/2015/159549

**Published:** 2015-11-12

**Authors:** Qian Ni, Feng Qi Wan, Yu Hong Jing, Xiang Yu Dong, You Cheng Zhang

**Affiliations:** ^1^Department of Pediatrics, Lanzhou University Second Hospital, Lanzhou 730000, China; ^2^Laboratory of Pediatrics, Lanzhou University Second Hospital, Lanzhou 730000, China; ^3^Institute of Anatomy, School of Basic Medicine, Lanzhou University, Lanzhou 730000, China; ^4^Department of General Surgery, Hepatic-Biliary-Pancreatic Institute, Lanzhou University Second Hospital, Lanzhou 730000, China

## Abstract

In this study, we aimed to investigate the effect of acute and chronic exposure to HA on the aerobic and anaerobic metabolism in liver by determining the hepatic levels of ICDH and ATP. Lactate levels in liver and blood were also examined. Rats were exposed to an altitude of 4,300 m for 30 days, and those without HA exposure were used as controls. We observed an increased expression of liver ICDH following acute exposure (days 1, 3, and 7), whereas the liver ATP concentration was reduced on day 1. No changes in the hepatic expression of ICDH and ATP were found in rats chronically exposed to HA. Lactate concentrations of liver and blood did not show any significant changes following HA exposure. Thus, aerobic metabolism may be the major metabolic pathway in response to HA hypoxia in order to acclimatize themselves to the stressful environments.

## 1. Introduction

Hypoxia-induced biochemical and metabolic changes are essential to the acclimation to the environment of lower oxygen such as high altitude (HA). The impact of acute and chronic exposure to HA on the aerobic and anaerobic metabolism has attracted a wide attention. Previous studies have reported that acute exposure to HA results in an anaerobic stimulation of glycolysis [1–6], and chronic HA acclimatization is characterized by an improved ability of efficient utilization of oxygen [1, 4, 6, 7]. However, other studies have suggested that the anaerobic metabolism remained unchanged during the whole HA exposure [8]. Apparently, the impact of acute and chronic HA acclimatization on the aerobic and anaerobic metabolism is still controversial.

Krebs cycle and fatty acid (FA) oxidation are oxygen-consuming processes; namely, they are aerobic metabolic pathways. Based on the previous studies, energy generation in animals exposed to HA environment may be through aerobic or anaerobic pathways. In our previous study, we found that FA oxidation in rats exposed to HA was increased during acute exposure and returned to the control group level during chronic exposure [9]. In this study, we aimed to evaluate the effect of acute and chronic HA exposure on Krebs cycle in rats.

ICDH, an important rate limited enzyme in Krebs cycle, is regarded as a potential target for altered metabolism under stress [10]. ATP is the end product in Krebs cycle. Liver is the most important organ in Krebs cycle and *β*-fatty acid (FA) oxidation. Thus, we examined the hepatic levels of ICDH and ATP concentration in rats exposed to 4300 m HA environment following 30 days. At the same time, we chose to examine the concentrations of lactate in liver and plasma as the indicators for anaerobic metabolism [7].

## 2. Materials and Methods

### 2.1. Animals

The detailed procedures for the animal studies have been described in our previous report [9]. Thirty-six male SD rats (body weights: 220–300 g) were kept at a density of 1 rat/cage and placed at an altitude of 400 meters at 22 ± 1°C. After 3 days of acclimatization, rats were randomly assigned into six groups, respectively, named H1 (*n* = 6, HA exposure for 1 day), H3 (*n* = 6, HA exposure for 3 days), H7 (*n* = 6, HA exposure for 7 days), H15 (*n* = 6, HA exposure for 15 days), H30 (*n* = 6, HA exposure for 30 days), and C (*n* = 6, no HA exposure, used as controls). For hypobaric hypoxic exposure, H1, H3, H7, H15, and H30 groups were air-transferred to a plateau experimental base (altitude 4,300 meters). Rats were housed at the same density at 22 ± 1°C in a humidity- and light-controlled room (lights on at 06:30 hours and off at 18:30 hours). Rats were given the standard rodent chow and water* ad libitum.* Following overnight fasting, rats were sacrificed under anaesthesia with 10% chloral hydrate (0.4 mL/100 g body weight, i.p.). Blood samples were collected into the heparinized tubes, and plasma was separated and stored at −80°C until analysis. The liver tissues were snap-frozen in liquid nitrogen and then stored at −80°C until analysis. The rats in Group C were anaesthetized and sacrificed on day 1 and processed in the same manner as described above. The study was approved by the Animal Care and Use Committee of the Lanzhou University.

### 2.2. Measurement of Liver ATP by Spectrophotometry

Liver ATP concentration was assayed by spectrophotometry using an assay kit (Nanjing Jiancheng Bioengineering Institute, Nanjing, China). The absorbance at 636 nm was recorded and the concentration of ATP was expressed as millimoles per gram tissue (mmol/g tissue).

### 2.3. Measurement of Lactate in Plasma and Liver by Spectrophotometry

As liver almost does not use lactate as energy source and lactate is a well-accepted marker for anaerobic metabolism [7], we measured the hepatic and plasma levels of lactate in the experimental rats to examine the impact of HA on the anaerobic metabolism. Hence, lactate concentrations in plasma and liver were assayed by spectrophotometry using an assay kit (Nanjing Jiancheng Bioengineering Institute, Nanjing, China). The absorbance at 530 nm was recorded. Frozen liver samples (50–100 *μ*g) were homogenized and the protein concentrations were measured by BCA Protein Assay Kit (Beyotime Institute of Biotechnology, Haimen, China). The concentration of lactate in plasma was expressed as millimoles per liter and that in liver tissues was expressed as millimoles per gram protein (mmol/g protein).

### 2.4. Hepatic Expression of ICDH in Rats Exposed to HA

In order to examine the impact of HA on the energy metabolism, we measured the hepatic expression of ICDH in the experimental rats at the mRNA level by quantitative real time polymerase chain reaction (qPCR) and the protein level by western blot, as we previously reported [9].

For qPCR studies, total RNA was extracted from the rat livers using RNAiso Plus reagent (TaKaRa Biotechnology Co., Dalian, China). Approximately 0.5 *μ*g of the extracted RNA was reverse-transcribed into cDNA using Primescript reverse transcription (RT) Master Mix (TaKaRa Biotechnology Co., Dalian, China). Reverse transcription reaction was performed at 37°C for 15 min followed by 85°C for 5 s. qPCR was performed in a final volume of 25 *μ*L, using the SYBR Premix Ex Taq II kit (TaKaRa Biotechnology Co., Dalian, China) on a Rotor-Gene 6000 Thermal Cycler. Each 25 *μ*L PCR reaction contains 2.0 *μ*L of cDNA, 1.0 *μ*L of sense primer, 1.0 *μ*L of antisense primer, 12.5 *μ*L of SYBR Green PCR Master Mix, and 8.5 *μ*L of the PCR-grade water. The cycling conditions are as follows: 95°C for 30 s, 40 cycles of 95°C for 5 s, and 60°C for 30 s. Each sample was assayed in duplicate. Fold inductions were calculated using the 2^−ΔΔCt^ method with *β*-actin being the internal reference. The primer sets used in this study were designed and synthesized by TaKaRa Biotechnology Company. ICDH primer sequence is 5′-GAGGCTTCATCTGGGCCTGTAA-3′ in sense and 5′-CATGGGCAGCCTCTGCTTCTA-3′ in antisense.

For protein expression by western blot analysis, frozen liver samples (50–100 *μ*g) were homogenized by manual grinding at 4°C in 200 *μ*L of RIPA lysis buffer (Beyotime Institute of Biotechnology, Haimen, China) supplemented with 1 mM PMSF. Insoluble material was removed by centrifugation for 10 min at 12,000 g at 4°C. The protein concentration in the supernatant was determined by BCA Protein Assay Kit (Beyotime Institute of Biotechnology, Haimen, China). Approximately, 50 *μ*g of the extracted protein sample from each animal was denatured in loading buffer, separated by 10% sodium dodecyl sulfate polyacrylamide gel electrophoresis (SDS-PAGE) at 30 mA constant current, and transferred to polyvinylidene difluoride (PVDF) membranes. The membranes were blocked with 5% skim milk in tris-buffered saline (TBS) containing 0.1% Tween (TBST) for 2 h at room temperature. Membranes were incubated with the primary antibodies (1 : 500) in TBST overnight at 4°C. Rabbit anti-*β*-actin (1 : 1000) was used to detect *β*-actin as the loading control. The membranes were then washed three times with TBST and incubated with a horseradish peroxidase- (HRP-) conjugated goat anti-rabbit IgG (1 : 10,000) for 2 h at room temperature. After two 10 min washes with TBST and one 10 min wash with PBS, the signals were detected using the enhanced chemiluminescence (ECL) substrate (Beyotime Institute of Biotechnology, Haimen, China) and imaged using an ImageQuant 350 Imaging System (GE Healthcare Bio-Sciences Corp., Piscataway, USA). All antibodies were purchased from BIOGOT Technology Co. (Nanjing, China). The western blot bands were analyzed by Image-Pro Plus Analysis Software Version 6.0 (Media Cybernetics, Inc., Rockville, MD, USA) and expressed as the relative integrated intensity compared to that of the *β*-actin of the same sample.

### 2.5. Statistical Analysis

All data are presented as means ± SD. The data were analyzed using one-way ANOVA with Tukey's test or Tamhane's T2 test for pairwise comparisons between the means. SPSS (version 17.0) and GraphPad (version 5) software were used for all statistical analysis. A *P* < 0.05 was considered to be statistically significant.

## 3. Results

### 3.1. Expression of ICDH and Concentration of ATP in Liver

**Figure 1:**
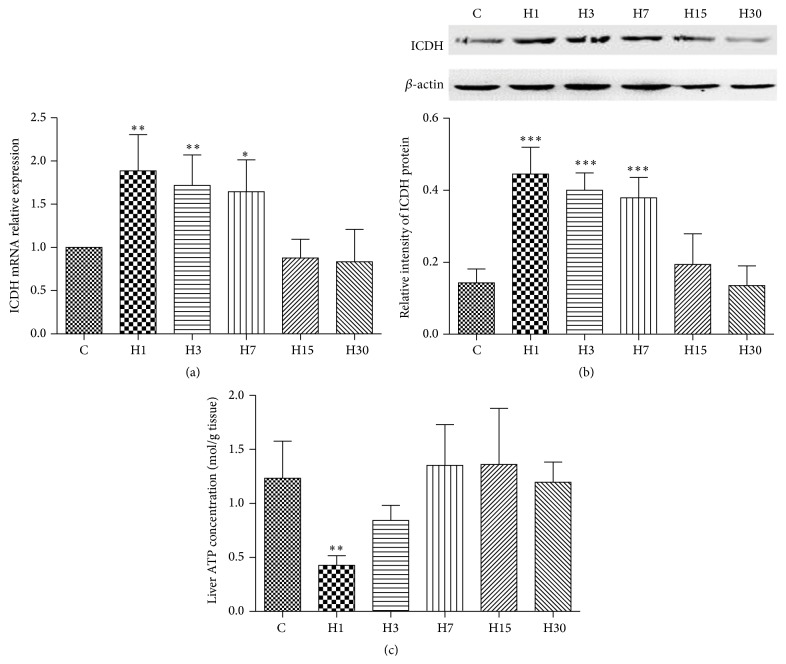
Relative expression of ICDH mRNA (a), ICDH protein (b), and liver ATP concentration (c) in rats exposed to HA. The expression of mRNA was measured by qPCR and the protein expression by western blot. The liver ATP concentration was measured by spectrophotometry. H1: rats were exposed to HA for 1 day; H3: rats were exposed to HA for 3 days; H7: rats were exposed to HA for 7 days; H15: rats were exposed to HA for 15 days; H30: rats were exposed to HA for 30 days. Control group: rats were not exposed to HA (*n* = 6). For ICDH mRNA analysis (a), *n* = 5 in H7 and H15, and *n* = 6 in H1, H3, and H30. For ICDH protein expression and liver ATP concentration analysis (c), *n* = 6 in each group. ^*∗*^ *P* < 0.05, ^*∗∗*^ *P* < 0.01, and ^*∗∗∗*^ *P* < 0.001.

To evaluate the change of glucose aerobic oxidation in the environment of HA, we measured the expression of ICDH and concentration of ATP in rat liver. Compared to control rats, significant acute increase in the expressions of ICDH mRNA was observed on 1, 3, and 7 days following HA exposure (1 versus 1.89 ± 0.42 in H1, 1 versus 1.72 ± 0.35 in H3, and 1 versus 1.64 ± 0.37 in H7, resp., *P* < 0.05). ICDH proteins have the same trend (0.45 ± 0.07 versus 0.14 ± 0.04 in H1, 0.40 ± 0.05 versus 0.14 ± 0.04 in H3, and 0.38 ± 0.06 versus 0.14 ± 0.04 in H7, resp., *P* < 0.001). After 15 days (H15), both mRNA and protein levels of ICDH were similar to that of the control (Figures 1(a) and 1(b)).

The above changes were associated with a significant decrease of the hepatic ATP level in rats exposed to HA for 1 day (H1) (0.43 ± 0.089 versus 1.23 ± 0.34, *P* < 0.01) (Figure 1(c)).

### 3.2. Concentration of Lactate in Plasma and Liver

**Figure 2:**
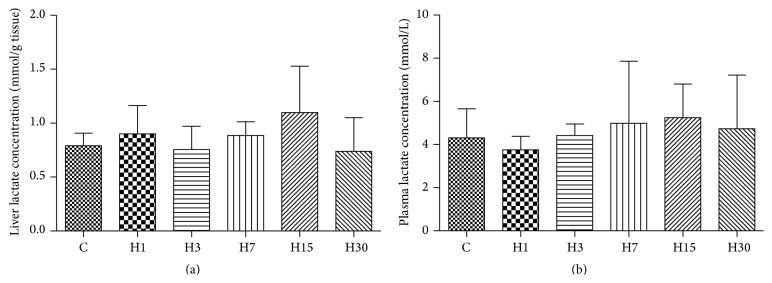
Lactate concentrations in the rat livers (a) and lactate concentrations in the rat plasma (b) were measured by spectrophotometry in control rats (no HA exposure, *n* = 6) and rats exposed to HA (*n* = 6).

Compared to control rats, no significant changes were found in the plasma and hepatic lactate concentration at all time points (Figures 2(a) and 2(b)).

## 4. Discussion

Altitude acclimatization is a process of adapting to hypobaric hypoxia. It is still a matter of debate whether aerobic or anaerobic metabolism is the major metabolic pathway in hypoxic conditions [1, 4, 6–8]. Both Krebs cycle and fatty acid (FA) *β*-oxidation are oxygen-consuming process, and ATP and ketone body are the major end products of these two pathways, respectively. ICDH is a key rate-limiting enzyme in Krebs cycle, and CPT-I is the key rate-limiting enzyme controlling FA *β*-oxidation, and the level of both enzymes may reflect the altered metabolism under stress [10–13].

In this study, we chose to examine several indicators to find out whether aerobic or anaerobic metabolism is the preferred metabolic pathway in rats exposed to HA condition. We first measured the hepatic expression of ICDH at the mRNA and protein levels. We noticed that, in rats exposed to acute HA condition, there was a significant increase in the expression of ICDH at the mRNA and protein levels (1, 3, and 7 days), and the levels were similar to those of the control rats in the chronic HA exposure groups. Meanwhile, the hepatic ATP concentration was reduced following acute HA exposure (1 day). We speculate that reduced ATP production would lead to increased expression of ICDH, which would in turn accelerate Krebs cycle and subsequently increase ATP production to the level of the control group. In our previous studies, we observed a significant increase of CPT-I at early acute exposure (3 days), a marked decrease of CPT-I at early chronic HA exposure (15 days), and a significant restoration of CPT-I at chronic exposure (30 days) [9]. In addition, we observed that increased mitochondrial *β*-oxidation of FAs in the liver leads to increased production of ketone body [9]. These data suggested that aerobic metabolism was likely the main pathway in the process of HA acclimatization. These findings also indicate that liver has a strong ability to adopt itself to hypoxia in order to maintain the essential level of energy generation.

In our study, we found no evidence of increased anaerobic metabolism, as reflected by the barely altered lactate level in both liver and blood of the animals exposed to HA condition.

It should be noted that our findings are not in complete agreement with the data published by others. For example, it was reported that, in mice exposed to acute HA (6 or 8 hours), there was a significant reduction in hepatic ICDH and an increase in plasma lactate level [2, 5]. Brooks et al. [1] have reported that, relative to the controls at the sea level, acute exposure of healthy men to 4,300 m led to 4-fold increase in the arterial lactate appearance rate (Ra). Dutta et al. [14] reported that, in rats exposed to acute HA (1 day), there was a significant reduction in the liver mitochondrial CPT-I, whereas slightly longer exposure (7 days) did not cause any significant changes. Lactate was found to be the major energy substrate in the animals exposed to HA, especially during the acute acclimatization [1, 2, 5, 6], suggesting that anaerobic metabolism plays a major role in the energy production under the acute HA condition. We speculated that such a partial discrepancy between the reported findings and our own data may reflect a difference in the experimental design. The HA exposure was simulated at an altitude of 8,000 m in Chen's study, 6,000 m in Liu's study, and 6,096 m in Dutta's study, whereas, in our experiments, rats were exposed to an altitude of 4,300 m. Thus, in the studies by other researchers, subjects were exposed to a much severe hypoxia. The experimental subjects would need to undergo very harsh adjustment in their metabolic system to meet their energy requirement. In the study by Brooks et al. [1], all study subjects were healthy males who were restricted to food intake but were given intravenous glucose before collecting blood samples. Such a different experimental design may likely explain the observed discrepancy between the said study and our own data.

In summary, increased aerobic metabolism may be one of the important mechanisms to respond to the HA-induced hypoxia during acute exposure in order to acclimatize themselves to the stressful environments. Aerobic metabolism is likely the main metabolic pathway during chronic exposure of HA. More studies are necessary to discover the underlying molecular mechanisms.

## Abbreviations

ICDH: Isocitrate dehydrogenase
CPT-I: Carnitine palmitoyltransferase-I
HA: High altitude
FA: Fatty acid.
